# Behind the times? Associations of working-time autonomy with health-related and occupational outcomes in health care personnel– a latent profile analysis

**DOI:** 10.1186/s12889-024-18289-0

**Published:** 2024-03-15

**Authors:** Franziska U. Jung, Alexander Pabst, Margrit Löbner, Melanie Luppa, Steffi G. Riedel-Heller

**Affiliations:** Institute of Social Medicine, Occupational Health and Public Health (ISAP), Faculty of Medicine, Leipzig University, Philipp-Rosenthal-Straße 55, 04103 Leipzig, Germany

**Keywords:** Working time, Satisfaction, Health, Turnover, Work ability

## Abstract

**Background:**

In the light of personnel shortage, the health care sector is facing the challenge to combine increasing employees‘ as well as patients’ needs. The aim of this study was to investigate the association between working-time autonomy and health-related (fatigue, psychosomatic complaints and work ability), as well as occupational outcomes (job satisfaction and turnover intention) in a large sample of health care employees.

**Method:**

Based on data of the BauA-Working Time survey, a sample of *n* = 1,093 employees working in the health care sector was analysed. Outcomes were assessed by the German Fatigue Scale, the Work Ability-Index and single-item measurements. Besides descriptive analyses, latent profile analysis (LPA) was used to determine clusters of employees based on working-time autonomy. Subsequently, regression analyses have been conducted to examine the association between autonomy clusters with health-related and occupational outcomes, controlling for sociodemographic characteristics and employment status.

**Results:**

LPA revealed that a three-cluster model was most suitable: high autonomy (cluster 1), medium autonomy (cluster 2) and low autonomy (cluster 3). The extracted profiles of working-time autonomy differed significantly in terms of sociodemographic and occupational characteristics, but not in terms of average working hours per week or monthly household income. The multivariate regression analysis revealed that being in the low-autonomy cluster was associated with more psychosomatic health complaints (IRR: 1.427, *p* = 0.008), lower work ability (OR 0.339, *p* < 0.001), as well as less job satisfaction (OR 0.216, *p* < 0.001).

**Discussion:**

Overall, the analyses indicate that it is crucial to prospectively consider working-time autonomy as an important factor of satisfaction, well-being and turnover intention in health care employees.

**Supplementary Information:**

The online version contains supplementary material available at 10.1186/s12889-024-18289-0.

## Introduction

### Importance of autonomy and flexibility– theoretical considerations

The basic idea of ​​the Job Demand-Control model [1] is to derive work-related stress from the combination of two dimensions of work content: the dimension of the quantity and nature of demands on the employee and the dimension of job control and workplace autonomy [2]. In this context, so-called high strain jobs, characterized by high demands and low levels ofautonomy, may contribute to the development of stress and result in negative health outcomes. Especially in the health care sector, employees are highly confronted with mental demands, increasing fatigue or emotional exhaustion [3]. At the same time, the growing needs of an aging population as well as the global shortage of health care professionals can be seen as additional challenges and demands. According to the Job-Demand-Control Model [1], control and autonomy related to working time schedules may be related to well-being and job satisfaction in health care professionals, depending on the level of demand.

### New work and working hour arrangements

In light of the establishing concepts of New Work [4], self-determination and autonomy have been the focus of recent studies. Among many different professions, personal and career needs have changed by shifting from traditional labour orientation towards work-life balance and changes in work-related values [5–7]. This is accompanied by innovative or flexible working time arrangements such as 4-day-weeksfour-day workweek, remote working, or being able to work flextime; however, recent studies confirm that working-time flexibility is often employer-oriented instead of employee-oriented [8, 9].

Autonomy and influence regarding working hour arrangements have become more and more important in explaining health complaints and dissatisfaction with working conditions [10–13].

### Empirical evidence of working time arrangements

In general, the duration and timing of work are important as they determine the possibility of recovery from work. However, if recovery does not take place or is insufficient, stress reactions accumulate into chronic impairments that are only partially reversible [14]. Previously, most studies focused on the length (i.e. part-time vs. full-time, overwork hours), as well as other dimensions such as shift work, working at night or on weekends, and the number of working days in a row. Since recovery may depend on individuals as well as work-related factors, it may be helpful for employees to be able to make their own decisions regarding their working-time arrangements (i.e., when to take breaks or timing of holidays). Based on a systematic literature review, another theoretical model has been suggested, which describes how working time arrangements may be related to health and well-being [15]. According to this model, factors directly related to working time (e.g., shift length and rotation) and factors unrelated to working time (i.e., length of recovery period, home life) may have synergistic effects on physiological processes (i.e., circadian disorders), leading to short- and long-term health effects in employees. Since working-time autonomy can affect both, working time and recovery phases, long-term health consequences (i.e., psychosomatic complaints or fatigue) can be assumed as a result of lacking autonomy with regard to working time. So far, low working-time autonomy has been associated with stress, poor health, and sleep disturbances across different professions [16, 17]. Another study found that time control reduced depressiveness in a sample of older employees [18]. Regarding health care professionals, being able to control the timing of breaks (and therefore recovery) and autonomy regarding starting and ending times have been linked to lower levels of work-life conflict and fewer sleep difficulties [11]. However, no clear associations were found regarding fatigue or perceived health. Interestingly, there is also evidence that working-time autonomy may not always be beneficial as autonomy may lead to employees being at risk for excessively extending their working hours [19, 20].

The topic of “control and autonomy” with regard to working hours is relatively new, especially in the health care system due to strict structural standards and patient needs that do not leave much room for self-determination and influence in the context of working hours. As mentioned above, there is evidence that working hour autonomy may be beneficial in terms of overall health and job satisfaction, but there is also evidence that a certain amount of autonomy may rather lead to excessive extension of working hours [20]. Moreover, different facets and degrees of working-time autonomy (such as timing of breaks or holidays or taking some days off) should be considered to gain comprehensive insights [16, 20].

The first aim of the present study was to identify different types of profiles of working-time autonomy among healthcare personnel using Latent Profile Analysis (LPA). As a second aim, we examined how these different profiles of working-time autonomy relate to important health-related and occupational outcomes, such as health complaints, fatigue, work ability, job satisfaction, and turnover intention. These outcomes may not only result in negative impacts on an individual level but may also affect the health care system in the long term. So far, there has been no study investigating the link between working-time autonomy and health-related and occupational outcomes in health care personnel. Investigating whether and how different degrees of autonomy may differently impact factors, such as job satisfaction or turnover intention, may close this gap. Based on these findings, working conditions may be altered in order to reduce personnel shortage and improve patient care in a variety of settings.

## Method

### Sample

The current study is based on data from the BAuA-Working Time Survey of the Federal Institute for Occupational and Health (BAuA) in Germany. The BAuA-Working Time Survey is a longitudinal study, including all employees with a minimum working time duration of at least ten hours a week (paid work). For the purpose of this study, we used factual anonymized data from the third wave, which was conducted in 2019 (Scientific Use File of the BAuA-Working Time Survey 2019, Version 2, [21]). Here, *n* = 10,540 employees took part in computer-assisted telephone interviews [22–24].

Employees below the age of 70 were included in the present study because individuals older than 70 were grouped together into one age group with no specification regarding the accurate age. Moreover and specifically in this study wave, participants working in health care settings were included in the analysis based on the following survey item: „*Do you work in: (i) home care; (ii) hospital/rehabilitation facility; (iii) retirement home/nursing home; (iv) day/night care center; (v) medical practice/day hospital; (vi) other*?“ (for more details, see Table A, supplementary file). The study sample included employed as well as self-employed participants, because being self-employed in health care may be associated with restricted working hour autonomy or independence (e.g., restricted opening hours for practices).

Information regarding sampling, interviewing, and consent has been described in detail elsewhere [22, 23]. In the third wave, the overall sample includes target persons from the previous waves (2015 and 2017) as well as a refresher sample of around 3,000 people in order to compensate for failures among the panel respondents and to reflect changes in the population. The telephone sample is based on a dual-frame approach and therefore relies exclusively on randomly generated landline and mobile phone numbers for the people surveyed for the first time. Data collection was carried out using computer-assisted telephone interviews (CATI). Participation in the BAuA Working Time Survey was voluntary, and therefore, a complete questionnaire acted as informed consent.

## Measures and instruments

In order to investigate the association between different dimensions of working-time autonomy and health-related and occupational outcomes in health care professionals, several instruments and items were used.

### Flexibility and autonomy of working time

Working-time autonomy was assessed with the following five items:

1. How much control do you have over the beginning or ending of your work day? (1 = very little control, 5 = very much control)
2. How much control do you have over when to make a break? (1 = very little control, 5 = very much control)
3. I manage to take my family and private interests into account when planning my working hours (1 = does not apply at all, 5 = fully applies).
4. How much control do you have over when to take a vacation? (1 = very little control, 5 = very much control)
5. How much control do you have on taking a few hours off from work? (1 = very little control, 5 = very much control)

### Health-related and occupational outcomes

We considered five working and health-related outcomes. First, fatigue was assessed using three items from the German Fatigue Scale [25]. A higher mean score reflects greater fatigue. One example item includes „I often lack energy.“ (answering categories: 1 = does not apply at all, 5 = does fully apply, Cronbachs‘ alpha: 0.78 ).

Second, participants were asked whether or not they were concerned with a variety of health complaints during the last 12 months (yes or no) [22]. These complaints included lower back pain, neck pain, headaches, sleeping difficulties at night, overall tiredness, gastro-intestinal issues, hearing impairment or tinnitus, irritability and tension, despondency, and physical and mental depletion. The presence of a health complaint was coded as “1” and summed up to a total score (i.e., the number of complaints).

In addition, job satisfaction was surveyed using a single item: „How satisfied are you with your job in general?“ (1 = very satisfied, 2 = satisfied, 3 = less satisfied, 4 = not satisfied) [26]. We further assessed turnover intentions with the following item: „I’ve been seriously considering changing employers for the past 12 months“ (answering format: 1 = does not apply at all, 5 = does fully apply) [22]. Finally, work ability was assessed using one item from the Work-Ability-Index [27]: „*If you rate your best ever work ability out of 10, how many points would you attribute to your current work ability? “0” means that you are currently unable to work*.”.

### Covariates

Apart from sociodemographic characteristics (e.g., age or gender), occupational characteristics (e.g., employment status or whether they work in an executive position) were part of this questionnaire. In addition, weekly working hours were assessed with the question, „*How many hours do you actually work per week, on average in this occupational activity, including regular overtime work, extra work, emergency services, etc.*?“.

### Data analysis

Latent profile analysis (LPA) was conducted to uncover distinct profiles of working-time autonomy among participants based on the five items of flexibility and autonomy of working time. LPA is a helpful strategy in order to identify distinct responses to a set of continuous items in work and organizational research [28, 29]. Compared to traditional, non-latent clustering, LPA has several advantages in general and with respect to the current study sample: (i) participants are grouped based on their (shared) probability of belonging to a certain cluster (estimated directly from the model); (ii) continuous and categorical variables can be used; and (iii) profile descriptions can be derived from sociodemographic characteristics or other (here: occupational) covariates [30]. Several indicators were used to determine the number of profiles with the best fit: The model with the optimal number of clusters has the lowest Bayesian information criterion (BIC)/sample size adjusted Bayesian information criterion (ssBIC) and the highest entropy, hence greater variability between the profiles, as well as the lowest Akaike information criterion (AIC) and G^2^ (deviance) [30–32].

Next, the distinct profiles obtained from the final LPA model were compared descriptively in terms of their sociodemographic and occupational characteristics, the distribution of working-time autonomy, and regarding the outcomes under investigation. Differences were tested using Kruskal Wallis equality of populations rank tests or Pearson’s Chi² test, when appropriate.

Univariate and multivariate models were applied in order to investigate the association between the different working-time autonomy profiles and the outcome variables. Due to the distribution of the outcomes “fatigue” and “psychosomatic health complaints,” generalized linear models (family: negative binominal, log-link function) were applied (reporting Incidence Rate Ratios and 95% confidence intervals). With regard to the outcomes “job satisfaction,” “turnover intention,” and “workability,” maximum likelihood estimation was performed (reporting odds ratios and 95% confidence intervals). All multivariate regression models were adjusted for sociodemographic (age, gender, education, income, childcare under the age of 18: yes/no) and occupational covariates (working hours per week, occupational status: employed/self-employed, shift work: yes/no, leading position: yes/no). Missing information regarding the variables of interest was lower than 1% (apart from income: 9.3%) and was handled by case-wise deletion in the regression analyses. All data were weighed to be representative of the German general population [22, 23]. The software program StataSE Version 16 has been used for all data analyses. Statistical significance was defined as *p* < 0.05.

## Results

Overall, the mean age of the study sample was 51 years, and the majority (74.7%) was female and employed (88.3%) rather than self-employed. The most common job areas were hospital/rehabilitation facility (*n* = 442), retirement home/care home (*n* = 287), and medical practice/day hospital (*n* = 277). Overall, 36.6% (*n* = 400) were working in shifts.

Table A (supplementary file) shows the results of the LPA using the working-time autonomy measures, including model fit indexes for n-cluster solutions. Based on the Bayesian-Information-Criterion (BIC) and entropy, the three-cluster model was found to fit the data best. The characteristics and differences of the three clusters will now be described in further detail in Table 1.

**Table 1:** Flexibility and autonomy regarding different dimensions of working time, M (SD)

	Cluster 1	Cluster 2	Cluster 3
Being able to influence when to start/end work (1 = very little control; 5 = very much control)	4.14 (SD: 1.41)	3.12 (SD: 1.22)	1.37 (SD: 0.79)
Being able to take some hours off (1 = very little control; 5 = very much control	4.79 (SD: 0.61)	3.24 (SD: 0.90)	1.56 (SD: 0.86)
Being able to influence timing of breaks (1 = very little control; 5 = very much control)	4.60 (SD: 0.93)	3.51 (SD: 1.05)	2.16 (SD: 1.30)
Being able to make allowance for private matters (1 = does not apply at all; 5 = does fully apply)	4.34 (SD: 0.91)	3.77 (SD: 0.79)	3.09 (SD: 1.25)
Being able to control the timing of vacations (1 = very little control; 5 = very much control)	4.58 (SD: 0.84)	3.86 (SD: 0.86)	3.35 (SD: 1.26)

*Note* M = mean; SD = standard deviation

With regard to the different facets of working-time autonomy, participants in cluster 1 show the highest degree of autonomy across all domains, whereas participants in cluster 3 show the lowest degree of autonomy (Table 1).

The three profiles are significantly different in terms of gender, education, and having children below 18 years of age (Table 2). No significant differences were obtained regarding the monthly household income of the participants. In addition, the three clusters did not differ in terms of their weekly working hours. However, cluster 3 included more shift workers, more employed participants compared to self-employed participants, and a greater number of participants in leading positions compared to the other two clusters.

**Table 2:** Sociodemographic and work-related characteristics of the overall study sample and the three clusteres

	Overall	Cluster 1– High autonomy	Cluster 2 Medium to high autonomy	Cluster 3 Low autonomy	Sign. Difference^1^
**Sociodemographic characteristics**					
Age	51.1 (SD: 9.7)	52.7 (SD: 8.9)	51.2 (SD: 9.5)	50.5 (SD: 9.5)	0.082
Gender					
female	816 (74.7%)	143 (72.2%)	290 (70.6%)	380 (79.2%)	0.009
Education < 12 years	542 (49.6%)	81 (40.9%)	178 (42.9%)	283 (59.0%)	< 0.001
≥ 12 years	547 (50.1%)	116 (58.6%)	237 (57.1%)	194 (40.4%)	
missing	4 (0.4%)	1 (0.5%)	/	3 (0.6%)	
Monthly income < 1000	76 (7.7%)	19 (10.9%)	22 (5.8%)	35 (8.1%)	0.091
1.001–2000	257 (25.9%)	41 (23.4%)	86 (22.5%)	130 (30.0%)	
2001–3000	259 (26.1%)	38 (21.7%)	88 (23.0%)	133 (30.7%)	
3001–4000	70 (7.1%)	29 (16.6%)	51 (13.4%)	22 (5.1%)	
4001–5000 > 5.001 missing	30 (3.0%) 102 (10.3%)				
children < 18 yrs, n (%) yes	340 (31.1%)	64 (32.3%)	150 (36.1%)	126 (26.3%)	0.006
**Work-related variables**					
Working hrs/week	36.3 (SD: 12.9)	35.7 (SD: 14.5)	36.7 (SD: 12.3)	36.1 (SD: 12.6)	0.353
Shift work yes	401 (36.7%)	25 (12.6%)	125 (30.1%)	251 (52.3%)	< 0.001
Occupational status being employed	965 (88.7%)	137 (69.9%)	364 (87.71%)	464 (97.3%)	< 0.001
Leadership position yes	642 (58.8%)	104 (52.5%)	222 (53.6%)	316 (65.8%)	< 0.001

*Note* M = mean, SD = standard deviation, monthly income = gross income, per person, in Euros; working hrs/week = including overtime hours; occupational status = being employed vs. self-employed; ^1^chi-square tests, ANOVA, and Kruskal Wallis, as appropriate

The distribution of health and job-related outcomes across the three clusters is summarized in Table 3, Figures A and B (see Supplementary File). According to the results, cluster 1 is characterized by the greatest job satisfaction (3.59), least psychosomatic complaints (3.05), the highest work ability (8.17) and the lowest turnover intention (1.80). In comparison, cluster 3 is characterized by the lowest job satisfaction (3.13), the highest psychosomatic complaints (4.88) and more fatigue (5.94), as well as the lowest work ability (7.40) and the highest turnover intention (2.47). The differences across the three clusters were all significant (see Table 3, Supplementary File).

**Table 3:** Distribution of health and job-related outcomes

	Cluster 1	Cluster 2	Cluster 3	Kruskal-Wallis equality of populations rank test
Job satisfaction (*n* = 1,093) (1 = very satisfied; 4 = unsatisfied)	3.59 (SD: 0.60)	3.33 (SD: 0.58)	3.13 (SD: 0.68)	x^2^(2) = 62.960, *p* < 0.001
Psychosomatic health complaints (multiple responses allowed)	3.05 (SD: 2.69)	3.68 (SD: 2.80)	4.88 (SD: 2.96)	x^2^(2) = 66.225, *p* < 0.001
Fatigue (*n* = 1,093), 0–15	4.66 (SD: 3.54)	4.65 (SD: 3.84)	5.94 (SD: 4.21)	x^2^(2) = 25.466, *p* < 0.001
Work ability (*n* = 1,092), 0–10	8.17 (SD: 1.62)	7.82 (SD: 1.81)	7.40 (SD: 2.18)	x^2^(2) = 21.885, *p* < 0.001
Turnover intention (*n* = 883) (1 = does not apply at all; 5 = completely applies)	1.80 (SD: 1.40)	1.91 (SD: 1.37)	2.47 (SD: 1.65)	x^2^(2) = 25.107, *p* < 0.001

*Note* M = mean, SD = standard deviation

In addition, univariate and multivariate regression analyses were performed to further investigate the associations between the working autonomy clusters and the outcomes (Table 4).

**Table 4:** Regression Analysis (not adjusted and adjusted for covariates)

	Fatigue (*n* = 1,093) IRR, 95% CI	Psychosomatic health complaints (*n* = 1,093) IRR, 95% CI	Work ability (*n* = 1,092) OR, 95% CI	Job Satisfaction (*n* = 1,093) OR, 95% CI	Turnover intention^1^ (*n* = 883) OR, 95% CI
**Unadjusted**					
Cluster 1: High autonomy Cluster 2: Medium autonomy Cluster 3: Low autonomy	Ref. 1.085 (0.852; 1.398) 1.315** (1.105; 1.565)	Ref. 1.572**(1.119; 2.207) 1.764***(1.421; 2.191)	Ref. 0.668 (0.357; 1.247) 0.431***(0.280; 0.663)	Ref. 0.232***(0.131; 0.411) 0.173***(0.094; 0.320)	Ref. 1.025 (0.475; 2.212) 2.480*(1.214; 5.063)
Wald Test	x^2^(2) = 10.52, *p* = 0.005	x^2^(2) = 26.40, *p* < 0.001	x^2^(2) = 14.76, *p* < 0.001	x^2^(2) = 33.80, *p* < 0.001	x^2^(2) = 13.07, *p* = 0.001
**Adjusted** ^**2**^					
Cluster 1: High autonomy Cluster 2: Medium autonomy Cluster 3: Low autonomy	Ref. 1.064 (0.845; 1.340) 1.233 (0.992;1.533)	Ref. 1.255 (0.958; 1.645) 1.427** (1.098; 1.855)	Ref. 0.521*(0.307; 0.886) 0.339***(0.185; 0.621)	Ref. 0.313***(0.164; 0.597) 0.216***(0.103; 0.450)	Ref. 0.688 (0.288; 1.648) 2.432 (0.993; 5.956)
Wald Test	x^2^(2) = 3.97, *p* = 0.137	x^2^(2) = 7.11, *p* = 0.029	x^2^(2) = 14.39, *p* < 0.001	x^2^(2) = 17.82, *p* < 0.001	x^2^(2) = 15.22, *p* < 0.001

*Note* *** *p* < 0.001; ** *p* < 0.010; * p < = 0.050; ^1^= employed participants only; 2 = models adjusted for gender, age, education, income, children under 18 years living in the same household, working hours/week, occupational status (employed vs. self-employed), leadership position (yes/no), shift work (yes); IRR = Incidence Rate Ratio; CI = Confidence Interval; OR = Odds Ratio

Regarding fatigue, participants in cluster 3 (low autonomy) show higher fatigue symptoms by an estimated 31.5% compared to cluster 1 (IRR 1.315, *p* = 0.002, unadjusted model). These effects diminished after introducing the covariates (adjusted model, Table 4).

With regard to the second health-related outcome, psychosomatic health complaints, results show that compared to cluster 1 (high autonomy), clusters 2 and 3 showed a significant change in number of health complaints (cluster 2: 57.2%, IRR: 1.572, *p* = 0.009; cluster 3: 76.4%, IRR: 1.764, *p* < 0.001). In other words, medium and low autonomy were related to more health complaints. After the introduction of covariates, the effect for cluster 3 remained significant (42.7%, IRR: 1.427, *p* = 0.008).

Furthermore, for each one-unit decrease in work ability, there were 2.32 higher odds in the low autonomy cluster (Cluster 3, OR: 0.431, *p* < 0.001, unadjusted model). Whereas in the multivariate regression model, we estimated 1.92 higher odds in the medium autonomy cluster (Cluster 2, OR 0.521, *p* = 0.016) and 2.95 higher odds in the low autonomy cluster (Cluster 3, OR 0.339, *p* < 0.001) for a one-unit decrease in work ability.

With regard to job satisfaction, there were 4.31 higher odds for a one-unit decrease in job satisfaction in the medium autonomy cluster (Cluster 2, OR 0.232, *p* < 0.001, unadjusted model) and 5.78 higher odds in the low autonomy cluster were estimated (Cluster 3, OR 0.173, *p* < 0.001). When the model was adjusted for covariates, these effects remained significant, with 3.19 higher odds in the medium (OR 0.313, *p* < 0.001) and 4.63 higher odds in low autonomy cluster (OR 0.216, *p* < 0.001 ) compared to the high autonomy cluster (Cluster 1).

The odds for turnover intention were as follows: Compared to cluster 1 (high autonomy), participants in the low autonomy cluster were 2.5 times more likely to have a higher turnover intention (Cluster 3, OR 2.480, *p* = 0.013). These effects diminished after introducing covariates (see Table 4, adjusted model).

## Discussion

The aim of the current study was to investigate the association between working-time autonomy and health-related and occupational outcomes in a broad sample of employees working within the healthcare sector. According to the latent profile analysis of the current study, three autonomy profiles were found. They are characterized by different degrees of working-time autonomy and vary in terms of health, work ability, satisfaction, and turnover intention.

These three types of autonomy can be described as “high autonomy,” “medium to high autonomy,” and “low autonomy” across all five domains of working-time autonomy that were included in the study. The results show that participants in the “high autonomy” cluster exhibit the highest values regarding job satisfaction and work ability, having fewer psychosomatic complaints and the lowest turnover intention. In contrast, participants in the “low autonomy” cluster are characterized by scoring lowest regarding job satisfaction and work ability but have the highest values of psychosomatic complaints and fatigue, as well as turnover intention.

The multivariate regression analyses confirm the association between autonomy and psychosomatic health complaints, work ability, and job satisfaction. Several aspects may explain these findings. The importance of working-time autonomy, especially being able to make allowances for private matters, in general has been shown before in a sample of physicians with different backgrounds [10]. In this context, work-life conflicts may contribute to the development of emotional exhaustion, which, after long-term exposure, may lead to chronic stress and burnout symptoms [33, 34]. As a result, this may cause a variety of health complaints and negatively impact work ability. In health care professionals, a “vicious cycle” can be assumed: reductions in work-life balance (due to low autonomy) may increase dissatisfaction and facilitate the intention to leave or early retirement [35–37]. Therefore, decreasing the remaining work force which will increase the quantity of work for those remaining within the health care system.

There are a few studies that have been able to prove a connection between working hours, health and job-related outcomes in health care professionals, as recently reviewed [38]. However, the topic seems complex, and the connection is influenced by a variety of factors, including age, family commitments, social skills, satisfaction with the medical field, physical activity, and sleep [38], which might explain why some effects in our study diminished after introducing covariates. Furthermore, being able to take some hours off or decide when to take a break may be important in determining job satisfaction and work ability, underlying the importance of individual recovery needs [39]. Besides positive effects on well-being and health, job satisfaction has also been the focus of several studies because it may determine work engagement, turnover intention, or retirement planning. In this context, it has been shown that working-time autonomy in general is positively related to job satisfaction, whereas scheduling autonomy moderates the relationship between working hours and job satisfaction [40, 41]. Even if working-time autonomy is not related to an employee’s performance, it can diminish the degree of exhaustion and work/non-work conflict [42]. Few studies with a focus on health care professionals have shown that increased autonomy regarding working hours (e.g., when to start or finish work, as well as the possibility of taking private interests into account within the planning of working hours) is associated with less workload and burnout, as well as fewer conflicts between work and private life, and increased job satisfaction [10, 11].

Even if the possibility of flexible, self-determined working hours has increased across other professional groups [22], this may not always be applicable within health care, as predetermined working time patterns (e.g., night shifts), especially in direct patient care or nursing, only allow some leeway. However, offering employees this kind of autonomy and influence may be a helpful strategy for attracting highly qualified personnel, especially in sectors with staff shortages. As an example, female employees may prefer flexible and autonomous working-time arrangements in order to handle the double burden of work and childcare. Future studies should find innovative working time schedules that offer employees within the healthcare sector this type of autonomy and, at the same time, allow for adequate and efficient patient care. In fact, research on work schedule design within healthcare or social assistance sectors has been ranked with the highest priority recently [43]. In this context, sociodemographic variability and, therefore, different needs should be focused on using further qualitative as well as quantitative study designs.

One limitation is the mean age of the study sample, as older employees were overrepresented. Therefore, important factors, such as caring for children, were only relevant to 31% of participants. However, previous studies have shown that flexible working hours are an important contributor to healthy aging at work, as perceived access to working hour flexibility was related to better health and psychological well-being in older employees [13, 44]. Besides, some outcome variables are based on single items. Therefore, it may be useful to replicate these results using validated or more optimal instruments in order to measure health-related and occupational outcomes.

In the current study, all concepts examined were operationalized via self-reports. This may lead to common method bias and should be taken into account when interpreting the results [45, 46]. However, self-reports have the ability to measure unobservables and are therefore useful in measuring subjective outcomes such as job satisfaction or influence on working time, as in the current study [47].

In addition to that, the results are based on cross-sectional data; therefore, no statements regarding causality can be made. Future analyses (e.g., based on the BauA-Working Time Survey follow-up) should approach the topic of working-time autonomy from a longitudinal perspective. However, it is rather unlikely that an employee’s job satisfaction may determine how much autonomy he or she is given by their employer. Working-time schedules are often predetermined by institutions or the job itself rather than the individual. Especially within the health care sector, where shift work or working on the weekend is very common, tight working-time arrangements can help to avoid operational disruption within patient care. Prospective studies could investigate whether it is autonomy per se (“I could if I wanted to”) or whether it is the combination of autonomy and actual determination that has positive effects on these outcomes. In other words, employees may be free to decide on their breaks or starting and ending times but cannot make use of them due to the high workload in patient care [11]. In addition, future studies may want to investigate the dynamic of processes and how changes in employees’ needs result in changes in these outcomes.

## Conclusion

The current study makes a significant contribution to the clarification of the association between working-hour autonomy and health-related and occupational outcomes in health care personnel. In-depth analysis using LPAs revealed that three types of working-hour autonomy exist which do not only differ in terms of sociodemographic characteristics but also in terms of job satisfaction, health, turnover intention and work ability. In the context of recent debates on how much flexibility and autonomy regarding working hours may be beneficial, the analyses indicate that it is crucial to consider autonomy as an important factor in satisfaction and well-being among health care employees; however, this should be done on an individual level. Institutions and employers need to find ways of establishing working-time autonomy in order to attract more personnel and keep the health care sector efficient and durable.

### Electronic supplementary material

Below is the link to the electronic supplementary material.

Supplementary Material 1

## Data Availability

The datasets used and/or analysed during the current study are available from the Federal Institute for Occupational and Health (BAuA) on reasonable request (Scientific Use File, contact: forschungsdaten@baua.bund.de).
