# Supplementary material for: Behind the times? Associations of working-time autonomy with health-related and occupational outcomes in health care personnel– a latent profile analysis
Source: BMC Public Health. 2024 Mar 15;24:825. doi: 10.1186/s12889-024-18289-0 (PMC10943857; doi:10.1186/s12889-024-18289-0)
Supplement: Supplementary file 1 — Supplementary Material 1 [file 12889_2024_18289_MOESM1_ESM.docx]

**Supplementary Material**


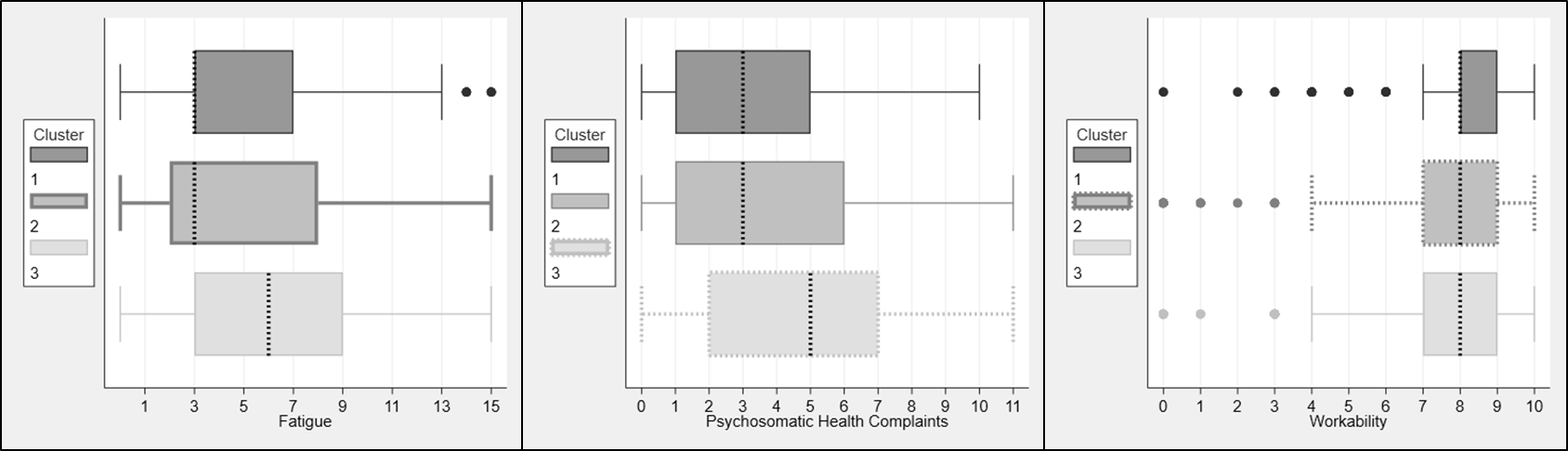


**Figure A: Cluster profile regarding health-related outcomes**

Note: Higher scores represent greater fatigue and work ability, as well as more psychosomatic complaints.


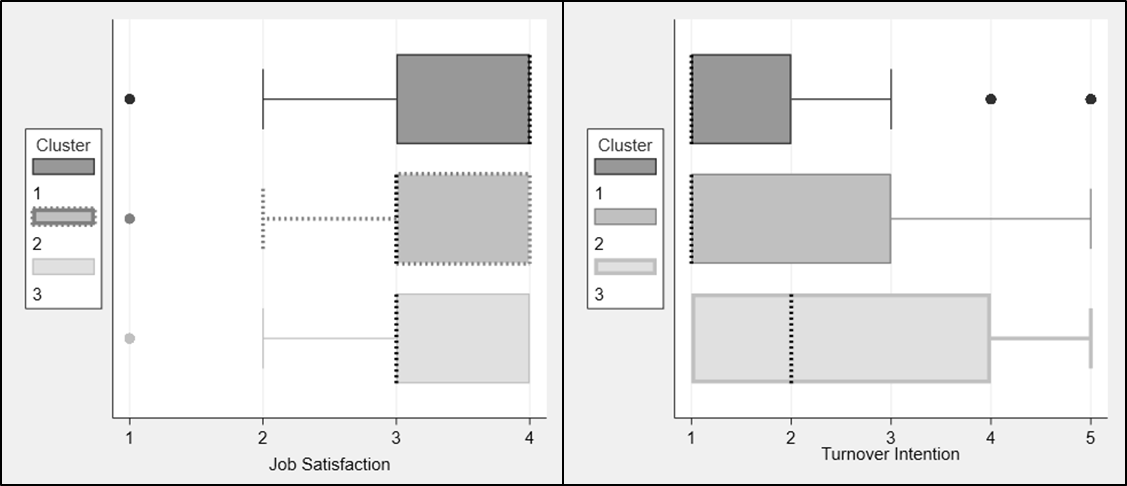


**Figure B: Cluster profile regarding occupational outcomes**

Note: Higher scores represent greater job satisfaction and greater turnover intention

**Table A: Model fit indexes**

|  | **AIC** | **BIC** | **SSBIC** | **Entropy** | **LogL** | **G^2^** | **Latent cluster marginal probablities**  **(margin, 95% conf. interval)** |
| --- | --- | --- | --- | --- | --- | --- | --- |
| **1 Cluster** | 16450.20 | 16550.13 | 16486.61 | 1.0 | -8205.10 | 2989.29 | / |
| **2 Cluster** | 15572.22 | 15777.08 | 15646.86 | 0.755 | -7745.11 | 2069.31 | 1 0.423 (0.372; 0.475)  2 0.577 (0.525; 0.627) |
| **3 Cluster** | 15231.92 | 15541.72 | 15344.79 | 0.760 | -7553.96 | 1687.01 | 1 0.180 (0.148; 0.216)  2 0.387 (0.335; 0.440)  3 0.434 (0.384; 0.485) |
| **4 Cluster** | 15174.21 | 15588.93 | 15325.30 | 0.725 | -7504.10 | 1588.00 | 1 0.357 (0.302; 0.415)  2 0.121 (0.057; 0.238)  3 0.355 (0.273; 0.445)  4 0.168 (0.134; 0.209) |

Note: AIC = Akaike information criterion; BIC = Bayesian information criterion; SSBIC = sample size adjusted Bayesian information criterion; LogL= log-likelihood; G^2^ = deviance
